# Structure-guided discovery and characterization of novel FLT3 inhibitors for acute myeloid leukemia treatment

**DOI:** 10.1371/journal.pone.0334415

**Published:** 2025-10-13

**Authors:** Bishal Budha, Gourab Basu Choudhury, Md. Shohag Hossain, Arjun Acharya

**Affiliations:** 1 Tri-Chandra Multiple Campus, Tribhuvan University, Kathmandu, Bagmati, Nepal; 2 CSIR-Indian Institute of Chemical Biology, Jadavpur, Kolkata, India; 3 Academy of Scientific and Innovative Research (AcSIR), Ghaziabad, Uttar Pradesh, India; 4 Department of Chemistry, Faculty of Science, University of Rajshahi, Rajshahi, Bangladesh; Institute of Medical Sciences, Banaras Hindu University, INDIA

## Abstract

FLT3 (FMS-like tyrosine kinase 3), a receptor tyrosine kinase, is frequently mutated in acute myeloid leukemia (AML), a hematologic malignancy marked by aggressive proliferation, poor prognosis, and high relapse rates. Although FDA-approved FLT3 inhibitors exist, their clinical efficacy is often undermined by resistance and off-target effects, underscoring the critical necessity for more effective and selective agents. Here, we employed a structure-based computational approach combining pharmacophore screening via Pharmit and the MolPort compound library to identify novel FLT3 inhibitors. Pharmacophore modeling, virtual screening, and docking identified two promising leads, MolPort-002-705-878 and MolPort-007-550-904, with binding affinities of –11.33 and –10.66 kcal/mol, correspondingly. These compounds were further evaluated using molecular dynamics (MD) simulations to assess binding stability, density functional theory (DFT) calculations to explore electronic reactivity, and ADMET profiling to examine pharmacokinetic and toxicity parameters. MD results, including principal component analysis (PCA) and free energy landscape (FEL) mapping, supported the integrity of the FLT3–lead complexes, with MM/GBSA binding free energies (ΔG) of –39.23 kcal/mol and –27.03 kcal/mol for MolPort-002-705-878 and MolPort-007-550-904, respectively. DFT analysis indicated favorable frontier molecular orbital energies and reactivity indices, characterized by a low HOMO–LUMO energy gap and a reactive dipole moment. ADMET predictions indicated acceptable drug-likeness and low toxicity, pending further experimental confirmation. This integrated *in silico* pipeline highlights the therapeutic potential of these molecules as next-generation FLT3 inhibitors and offers a scalable strategy for targeted AML therapeutics.

## Introduction

Acute myeloid leukemia (AML) is a fast-progressing blood cancer marked by the unchecked proliferation of immature myeloid cells in the bone marrow and bloodstream [1]. This clonal expansion disrupts normal blood cell production, resulting in bone marrow failure and swift decline in patient health [1,2]. Representing the most recurrent type of acute leukemia in mature individuals, AML accounts for over half of all adult leukemia cases and is associated with particularly poor outcomes in high-risk and elderly populations, where five-year survival rates remain below 30% [3,4]. Despite advances in identifying key genetic mutations such as FLT3, IDH1/2, and NPM1, and the curative potential of bone marrow transplantation in select patients, AML remains largely incurable, particularly in cases of relapse or treatment resistance. Moreover, AML is known for its rapid progression, and if left untreated, it can become increasingly aggressive, further emphasizing the urgent need for more effective and durable therapeutic strategies [5–7].

FMS-like tyrosine kinase 3 (FLT3) is a receptor tyrosine kinase predominantly expressed in hematopoietic stem cells and plays a pivotal role in the regulation of hematopoiesis [5]. Mutations in FLT3, particularly internal tandem duplications (FLT3-ITD) and tyrosine kinase domain point mutations (FLT3-TKD), are among the most frequent genetic abnormalities in acute myeloid leukemia, affecting nearly 30% of patients [8,9]. As a result of these mutations, the kinase remains persistently active, which promotes malignant signaling and is associated with adverse prognosis [10]. Consequently, FLT3 has gained recognition as a proven biological site in the therapeutic management of AML.

FLT3 inhibitors have been established as promising therapeutic agents for AML, supported by a wide array of preclinical and clinical studies [11–13]. FDA-approved inhibitors like Midostaurin, Quizartinib, and Gilteritinib exert their activity by targeting the ATP-binding cavity within the FLT3 receptor, thereby preventing its phosphorylation and downstream signaling [13–15]. Structurally, these inhibitors share bulky, heteroaromatic, polycyclic frameworks: Gilteritinib features a pyrazine-carboxamide core, Quizartinib employs a heteroaryl–aryl urea scaffold, and Midostaurin is derived from an indolocarbazole–pyrrolidone fused framework related to staurosporine analogs. Although all of these inhibitors have gained regulatory approval and are commercially available in the United States, their clinical effectiveness is often compromised by issues such as acquired resistance, off-target effects, and suboptimal specificity [14,16]. These limitations not only reduce therapeutic efficacy but also affect patient safety and quality of life. Consequently, there remains a pressing need for next-generation FLT3 inhibitors featuring novel chemical scaffolds with improved potency, selectivity, and pharmacokinetic profiles to achieve better clinical outcomes in AML therapy.

Structure-based virtual screening (SBVS) is a key component of modern CADD (Computer-Aided Drug Design), enabling the identification of candidate molecules based on their structural compatibility with the target binding site [17]. Among available platforms, Pharmit offers distinct advantages through its interactive, cloud-based interface that integrates pharmacophore modeling, shape screening, and energy minimization [18]. It allows real-time refinement of pharmacophore queries directly from protein-ligand complexes and supports rapid filtering of large compound libraries based on steric and electronic features [18].

Molecular docking estimates the most favorable binding pose and interaction strength of ligands at receptor sites, making it a key component of structure-based drug design [19]. However, its static nature limits understanding of dynamic interactions. To address this, molecular dynamics (MD) simulations are carried out to examine temporal stability, conformational mobility, and ligand persistence under near-physiological conditions [20]. Post-MD MM/GBSA calculations refine hit selection by quantifying binding free energies, accounting for solvation and entropic contributions [21]. Density functional theory (DFT) provides deep insights into electronic properties such as frontier orbitals and charge distribution, aiding in reactivity and binding specificity analysis [22]. Finally, *in silico* ADMET profiling evaluates pharmacokinetics and toxicity, ensuring drug-likeness and safety of the candidate compounds [23].

**Fig 1:**
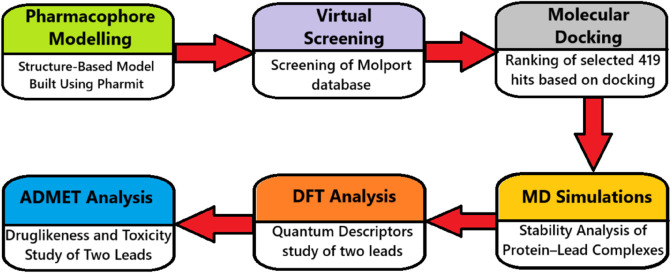
Workflow diagram providing a concise overview of the study. The figure illustrates the overall methodology and structure of the study, highlighting key stages.

This study presents an integrated computational strategy comprising pharmacophore modeling (via the Pharmit platform), molecular docking, MD simulations with PCA and FEL analysis, DFT calculations, and ADMET evaluation to guide the rational design of FLT3 inhibitors (Fig 1). By combining these complementary methods, the approach aims to systematically identify and characterize hit compounds with strong structural compatibility, binding stability, favorable electronic properties, and suitable pharmacokinetics. The eventual objective is to discover novel chemical scaffolds with strong potential to inhibit FLT3, advancing targeted therapies for acute myeloid leukemia.

## Materials and methods

### Pharmacophore modeling and virtual screening

In this study, we examined the crystallographic conformation of the FLT3 kinase complexed with the FDA-approved drug, Gilteritinib (PDB ID: 6JQR) [24], using it as a reference to identify key molecular interactions and define relevant pharmacophoric features. A ligand-based pharmacophore model was subsequently developed from this FLT3 crystal structure using the Pharmit web server [18].

This pharmacophore was employed to screen the MolPort compound library, one of the most recently curated and expansive repositories, containing 4,742,020 compounds as of September 5, 2024. All candidates were filtered according to Lipinski’s Rule of Five [25] for ensuring drug-likeness. Screening was performed with only the best conformer per compound to maintain computational efficiency. From the resulting dataset, the top 25,000 hits were selected, followed by energy minimization within the server to refine ligand geometries. Compounds exhibiting a maximum minimized binding affinity (docking score) of zero were retained. Finally, a threshold value of 1 Å for molecular root-mean-square deviation (mRMSD) was enforced, and compounds showing substantial deviation from the pharmacophore alignment were excluded, ensuring high fidelity between the screened conformations and the original pharmacophore model.

### Preprocessing of ligands and protein

The X-ray crystallographic structure of the FLT3 receptor (PDB ID: 6JQR) [24] was retrieved from the RCSB Protein Data Bank [26]. Homology modeling was performed using MODELLER to fill in missing residues and generate a structurally complete protein model [27]. Protein preparation was conducted using AutoDockTools [28], during which all water molecules were deleted, polar hydrogens were introduced, and Kollman partial charges were applied. Three-dimensional structures of the identified hits from pharmacophore-based virtual screening were imported from the screening server. Following energy minimization and charge assignment, PDB format files of both protein and ligands were converted into PDBQT format using OpenBabel [29].

### Molecular docking

The receptor grid for docking was constructed around key catalytic residues of FLT3; Leu616, Val624, Ala642, Glu692, Cys694, Asp698, and Leu818 based on their involvement in the active site [30], using grid dimensions of 20 Å, 25 Å, and 22 Å with a grid point spacing of 1 Å along the *x*, *y*, and *z* axes, respectively. Docking-based virtual screening was performed within that receptor grid using PyRx where Autodock Vina serves as the backend [29]. The cognate ligand was redocked into its binding site, and the RMSD between its crystallographic and predicted poses was calculated to validate the docking procedure. The resulting top protein–ligand complexes were visually examined using PyMOL 2.5.2 [31], molecular interactions were analyzed in BIOVIA Discovery Studio [32], and Tanimoto similarity to FDA-approved FLT3 inhibitors was computed using RDKit [33].

### Molecular dynamics simulations

GROMACS 2025.1 [34] was employed to conduct molecular dynamics simulations aimed at probing the structural stability of the protein and its ligand-bound forms [21]. The protein was parametrized deploying CHARMM General Force Field (CGenFF), and ligand topologies were generated via SwissParam [35]. Structures underwent energy minimization for 2500 steps using the steepest descent algorithm. The systems were solvated using the TIP3P water model [36] and neutralized with Na^ + ^ and Cl^−^ ions using gmx genion. Two equilibration phases followed: a 100-ps NVT run to reach 300 K, and a 100-ps NPT run for pressure and density stabilization. During equilibration, all bond lengths were constrained to preserve structural integrity, and water constraints facilitated solvent shell reorganization. The V-rescale thermostat and Parrinello–Rahman barostat [37] were employed for temperature and pressure control, respectively. The LINCS algorithm ensured bond constraints [38], and long-range electrostatics were calculated using the PME method [39]. Production MD simulations were executed for 100 ns to trace trajectory and stability analysis.

### MM/GBSA binding free energy

Binding free energies were calculated using the Molecular Mechanics/Generalized Born Surface Area (MM/GBSA) method via the gmx_MMPBSA tool (version 1.6.3), which integrates GROMACS with AmberTools for end-state energy analysis [40]. The overall binding free energy ($\Delta G_{\text{bind}}$) was partitioned into van der Waals ($\Delta E_{\text{vdW}}$), electrostatic ($\Delta E_{\text{ele}}$), and both polar and non-polar solvation energy contributions, as outlined in Eq (1) [21].

$$\Delta G_{bind}=\Delta E_{vdW}+\Delta E_{ele}+\Delta G_{polar}+\Delta G_{nonpolar}$$ (1)

Polar solvation energy was evaluated by the Generalized Born (GB) using the GB-Neck2 implicit solvent model (igb=5), and the nonpolar term was estimated via the LCPO method [41]. Entropy contributions were omitted, consistent with common practice in comparative MM/GBSA studies [42]. A total of 1001 snapshots were extracted at equal intervals from the 100 ns production trajectory (MD_center.xtc) for free energy analysis.

### Principal component analysis (PCA)

Trajectory pre-processing and PCA execution were performed using GROMACS 2025.1 [34]. The positional atomic covariance matrix was computed using the gmx covar command, focusing on the C*α* atoms of the protein to capture backbone motions. This step produced eigenvectors along with their corresponding eigenvalues. Subsequently, the top three principal components were then analyzed using the gmx anaeig utility to examine root-mean-square (RMS) fluctuations, eigenvector contributions, and 2D trajectory projections. All PCA plots were generated using Xmgrace [43].

### Free energy landscape (FEL)

The trajectory file from the 100 ns molecular dynamics simulation was taken as input for principal component analysis (PCA), from which the top two eigenvectors (PC1 and PC2) were selected as reaction coordinates. The free energy of each conformation was calculated on the basis of free energy ΔG, Boltzmann constant ($k_{B}=1.380649\times 10^{-23}$ J/K), absolute temperature (T = 298.15 K) and natural logarithm of the normalized probability distribution across the PC1–PC2 space (lnP), using the Boltzmann relation as shown in Eq (2) [44].

$$\Delta G=-k_{B}T\ln P$$ (2)

The three-dimensional FEL was visualized using the matplotlib.pyplot.plot_ surface() function to map local energy minima and transition states on a rugged conformational landscape [45].

### DFT analysis

The molecular structure and Gaussian input files of the leads were generated with the GaussView 6 program [46]. The molecular geometries of the leads were fully optimized in the gas phase employing density functional theory at the B3LYP level and the 6-311G(d,p) basis set, facilitated by the Gaussian 09W software package [47]. Optimized molecular structures were used to compute various molecular properties, including frontier molecular orbitals (HOMO and LUMO), the energy gaps (ΔE), quantum chemical descriptors, molecular electrostatic potential (MEP) map, Mulliken charges and natural bond orbital (NBO) studies. Key global reactivity descriptors such as ionization potential (*I*), electron affinity (*A*), chemical hardness (*η*), chemical softness (*S*), chemical potential (*μ*), global electrophilicity index (*ω*) and, nucleophilicity index (N) were derived from orbital energy values on the basis of Koopmans’ principles (Eqs (3) and (4)) [48,49].

$$I=-E_{\text{HOMO}},\;A=-E_{\text{LUMO}},\;\eta =\frac{1}{2}(I-A)$$ (3)

$$S=\frac{1}{\eta },\;\mu =-\frac{1}{2}(I+A),\;\omega =\frac{\mu^{2}}{2\eta },\;N=\frac{1}{\omega }$$ (4)

The NBO program [50] was employed to investigate natural bond orbitals at the same level of theory, where delocalized interactions were analyzed using second-order perturbation theory. The stabilization energy *E*(2) was computed from the donor orbital occupancy (*q*_*i*_), donor and acceptor energies ($E_{i},E_{j}$), and the Fock matrix off-diagonal element (*F*_*ij*_) as per Eq (5) [51,52].

$$E(2)=\frac{q_{i}F(i,j{)}^{2}}{E_{j}-E_{i}}$$ (5)

### ADMET analysis

The compounds with the most favorable docking scores were further evaluated for their pharmacokinetic properties using the SwissADME web server [23], and for toxicity using the ProTox-3.0 web server [53].

## Results and discussion

### Pharmacophore-based virtual screening

**Fig 2:**
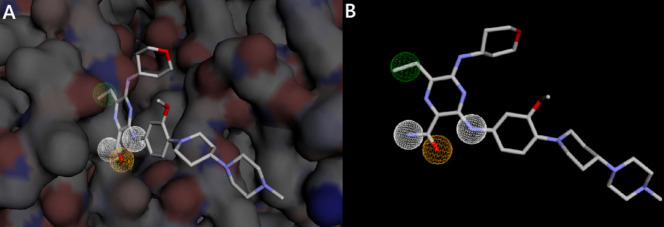
Pharmacophore features generated by the Pharmit server. A: Pharmacophore features mapped within the receptor binding pocket. B: Pharmacophore features mapped onto the ligand in the absence of the receptor structure. White spheres represent hydrogen donor regions, orange indicates hydrogen acceptor, while green indicates hydrophobic feature.

The pharmacophore query model constructed using the key interaction features of Gilteritinib observed within the FLT3 binding site (Fig 2 and S1 Fig) incorporated four critical pharmacophoric features: one hydrophobic region, one hydrogen bond acceptor, and two hydrogen bond donor groups, each of which was deemed essential for a compound to be considered a valid hit. The precise spatial coordinates (X, Y, and Z) of these features are detailed in Table 1. Utilizing this defined feature set, a ligand-based virtual screening was performed against the MolPort compound library and only those molecules matching all four features were retained. From the initial top 25,000 compounds identified as potential hits by the server, 419 compounds met the screening criteria: compliance with Lipinski’s Rule of Five, minimized binding affinity less than zero, and a molecular RMSD (mRMSD) below 1 Å. These 419 hits were selected (S1 Table) and transferred for further validation and refinement through docking-based virtual screening.

**Table 1:** Pharmacophore features and their computed 3D coordinates from Pharmit.

Parameter	Hydrogen donor (1)	Hydrogen donor (2)	Hydrogen acceptor	Hydrophobic
**X**	–28.282	-27.437	–27.632	-28.146
**Y**	–7.483	–3.380	–5.511	–2.242
**Z**	–30.381	–31.714	–32.244	–27.314
**Radius**	1	1	1	1

### Docking-based virtual screening

A total of 419 hits from pharmacophore modeling were subjected to molecular docking with FLT3 within the defined receptor grid. The resulting docking scores spanned from –11.330 to –3.052 kcal/mol. Based on these results, the top seven highest-ranking compounds crossing binding energy below –9 kcal/mol were shortlisted with their IUPAC names and binding energies in Table 2. The binding affinity profiles of these ligands suggest a significant potential to suppress the functional activity of the target protein.

**Table 2:** Binding energies and IUPAC names of the top selected compounds.

Compounds	IUPAC Name	Binding Energy (kcal/mol)
**MolPort-002-705-878**	N’-[(3E)-6-bromo-5-methyl-2-oxo-2,3-dihydro-1H-indol-3-ylidene]-3-hydroxynaphthalene-2-carbohydrazide	–11.33
**MolPort-007-550-904**	3-hydroxy-N’-[(3E)-2-oxo-1-propyl-2,3-dihydro-1H-indol-3-ylidene]naphthalene-2-carbohydrazide	–10.66
**MolPort-002-251-242**	2-chloro-5-([(5E)-1-ethyl-2,4,6-trioxo-1,3-diazinan-5-ylidene]methylamino)benzoic acid	–10.46
**MolPort-002-603-739**	(5E)-5-[(2-aminophenyl)amino]methylidene-1-(butan-2-yl)-2-sulfanylidene-1,3-diazinane-4,6-dione	–10.23
**MolPort-000-431-547**	1-(3,4-dichlorophenyl)-3-[2-oxo-5-(trifluoromethyl)-1,2-dihydropyridin-3-yl]urea	–9.52
**MolPort-002-602-969**	(5E)-5-(1-[4-(dimethylamino)phenyl] aminopropylidene)-1-(prop-2-en-1-yl)-1,3-diazinane-2,4,6-trione	–9.41
**MolPort-002-599-502**	(5E)-5-[(2-aminophenyl)amino]methylidene-1-cyclopropyl-2-sulfanylidene-1,3-diazinane-4,6-dione	–9.25

**Fig 3:**
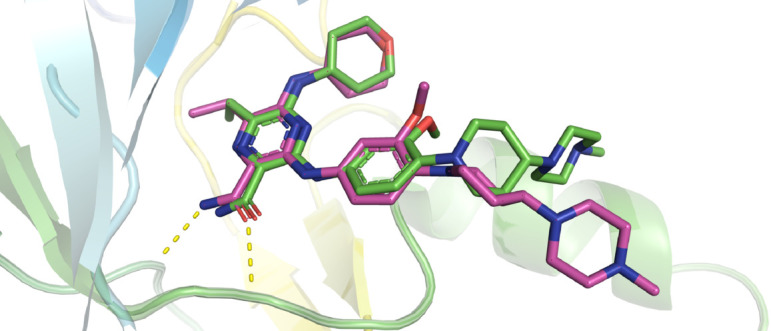
Superposition of native ligand and best-docked conformation. Green denotes the native ligand, whereas pink depicts the best-docked pose. The overlay yielded an RMSD of 0.72 Å, thereby validating the accuracy of the docking protocol.

Docking validation was performed by re-docking the cognate ligand Gilteritinib into its native binding zone. The resulting RMSD of 0.72 Å (Fig 3), which is well below the 2 Å threshold, confirms accurate reproduction of the experimental pose [54]. This establishes the robustness and predictive reliability of the docking workflow.

### Molecular interaction analysis

**Fig 4:**
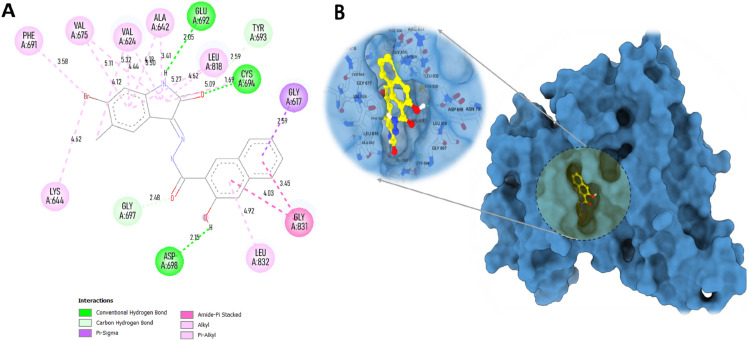
Interaction plots of MolPort-002-705-878 with FLT3 receptor. A: 2D interaction diagram. B: 3D interaction surface within the active site.

The top two compounds, MolPort-002-705-878 and MolPort-007-550-904 with binding affinities of –11.33 kcal/mol and –10.66 kcal/mol, correspondingly (Table 2), were selected as leads for detailed interaction analysis. MolPort-002-705-878 exhibited multiple key interactions within the active site. It formed four conventional hydrogen bonds with CYS694 (1.69 Å), ASP698 (2.15 Å), GLU692 (2.05 Å), and GLY697 (2.48 Å) as shown in Fig 4 and Table 3. A carbon–hydrogen bond was observed with GLY617 (2.59 Å), along with a pi–sigma interaction with LEU818 (4.62 Å) and an amide–pi stacked interaction with GLY831 (4.03 Å). Hydrophobic interactions included pi-alkyl contacts with LEU832 (4.92 Å) and GLY831 (3.45 Å), and alkyl interactions involving VAL624 (5.11, 5.32 Å), VAL675 (4.12 Å), ALA642 (4.30 Å), LEU818 (5.09, 4.62 Å), PHE691 (3.58 Å), and LYS644 (4.62 Å). These interactions stabilize the ligand within the binding pocket, highlighting its high affinity, with the presence of multiple hydrogen bonds further contributing to its enhanced binding energy [55].

**Table 3:** Interaction analysis of two lead compounds with FLT3 protein.

Compound	Interaction type	Interacting amino acids (Distance in Å)
**MolPort-002-705-878**	Conventional hydrogen bond	CYS694 (1.69), ASP698 (2.15), GLU692 (2.05), GLY697 (2.48)
	Carbon hydrogen bond	GLY617 (2.59)
	Pi-sigma	LEU818 (4.62)
	Amide-Pi stacked	GLY831 (4.03)
	Pi-Alkyl	LEU832 (4.92), GLY831 (3.45)
	Alkyl	VAL624 (5.11, 5.32), VAL675 (4.12), ALA642 (4.30), LEU818 (5.09, 4.62), PHE691 (3.58), LYS644 (4.62)
**MolPort-007-550-904**	Conventional hydrogen bond	ASP698 (2.19, 2.21), CYS694 (2.77), GLU692 (1.77), GLY697 (2.45)
	Carbon hydrogen bond	LEU832 (2.56)
	Pi-Pi T-shaped	PHE691 (4.91)
	Amide-Pi stacked	GLY831 (3.69), PHE691 (5.19)
	Pi-Alkyl	VAL675 (5.20), VAL624 (5.03), ALA642 (4.46), LEU818 (4.82)
	Alkyl	LEU616 (3.70), GLY831 (3.69)

**Fig 5:**
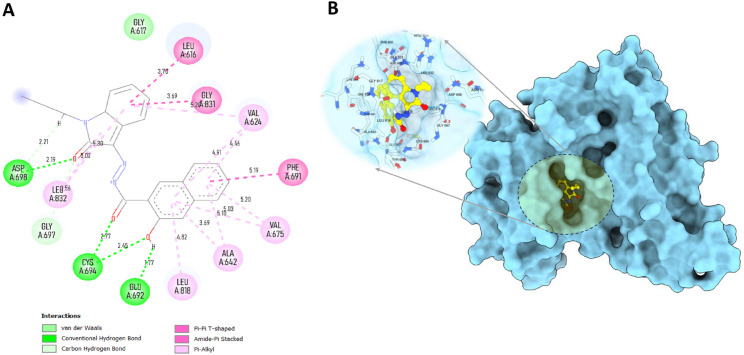
Interaction plots of MolPort-007-550-904 with FLT3 receptor. A: 2D interaction diagram. B: 3D interaction surface within the active site.

MolPort-007-550-904 also displayed significant interactions within the active sites (Fig 5), forming conventional hydrogen bonds with ASP698 (2.19, 2.21 Å), CYS694 (2.77 Å), GLU692 (1.77 Å), and GLY697 (2.45 Å) (Table 3). A carbon-hydrogen bond was formed with LEU832 (2.56 Å). Pi-pi T-shaped stacking was observed with PHE691 (4.91 Å), and amide-pi stacked interactions were detected with GLY831 (3.69 Å) and PHE691 (5.19 Å). Pi-alkyl interactions were found with VAL675 (5.20 Å), VAL624 (5.03 Å), ALA642 (4.46 Å), and LEU818 (4.82 Å), while alkyl interactions involved LEU616 (3.70 Å) and GLY831 (3.69 Å). Collectively, these interactions suggest a strong and specific binding orientation in the active site and contend for potent inhibitor of FLT3.

Both MolPort-002-705-878 and MolPort-007-550-904 share an ortho-hydroxynaphthalene-1-carboxamide (o-HNCA) motif, absent in FDA-approved FLT3 inhibitors (S2 Fig). This unit is conjugated to the exocyclic imino nitrogen of an imesatin (3-imino-2-oxindole) core. The first lead features vicinal 6-bromo and 7-methyl substitutions on the imesatin scaffold, while the second contains an N-propyl group at the lactam nitrogen and lacks aryl halogenation, indicating structural divergence. These features produce a compact, rigid, H-bond–competent benzolactam–amide framework distinct from clinically validated FLT3 chemotypes. Consistently, both leads showed low 2D similarity (Tanimoto <0.20) to FDA-approved FLT3 inhibitors, reinforcing scaffold-level novelty and providing a quantitative basis for their structural distinctiveness (S3 Fig).

### MD simulations

Among the seven top-ranked docked compounds, MolPort-002-705-878 and MolPort-007-550-904 were selected as leads based on favorable docking scores, consistent hinge-region hydrogen bonding (CYS694, GLU692, ASP698), and key hydrophobic interactions, which appeared more pronounced compared to the other shortlisted compounds. Molecular dynamics simulations confirmed the stability of these interactions, while MM/GBSA binding energies indicated strong binding affinity. Both compounds also exhibited acceptable ADMET profiles and favorable DFT-based electronic descriptors. These multi-parameter criteria supported their advancement for further structural and energetic evaluation.

**Fig 6:**
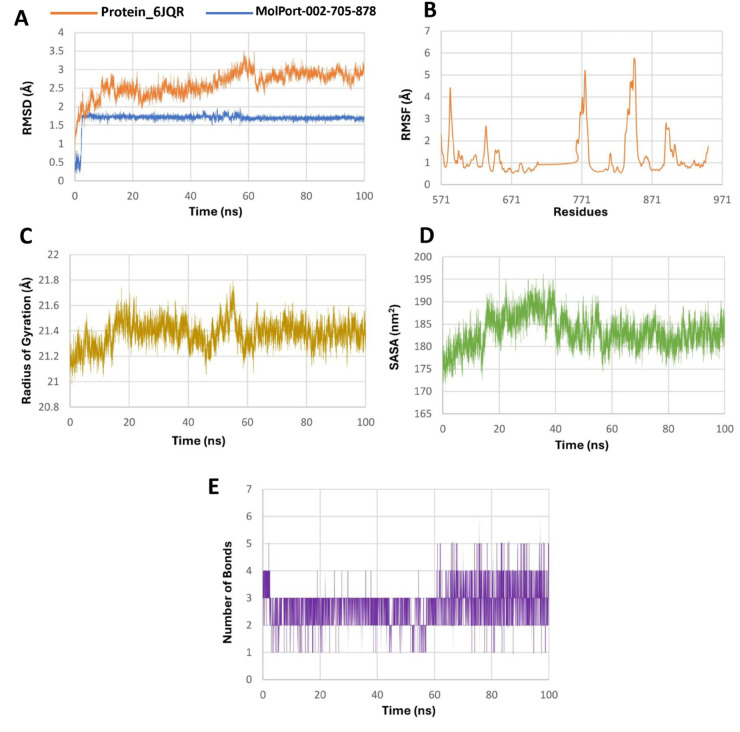
Molecular dynamics simulation analysis of the 6JQR–MolPort-002-705-878 complex over 100 ns. A: RMSD plot comparing structural stability of the protein (orange) and ligand (blue). B: RMSF plot showing residue-specific flexibility of the protein, with higher fluctuations in loop regions. C: Radius of gyration (Rg) indicating consistent compactness of the protein. D: SASA plot reflecting solvent accessibility changes during the simulation. E: Hydrogen bonding dynamics over time showing consistent ligand–protein interactions, reaching up to six bonds at peak points. These results suggest strong and stable ligand binding with enhanced interaction stability.

**Fig 7:**
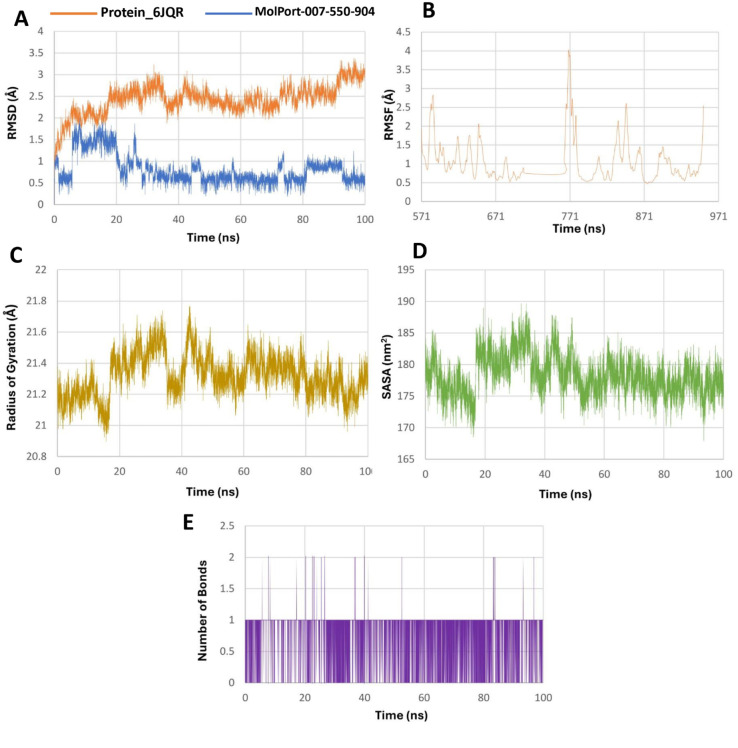
Molecular dynamics simulation analysis of the 6JQR–MolPort-007-550-904 complex over 100 ns. A: RMSD plot showing backbone stability of the protein (blue) and ligand mobility (orange). B: RMSF plot representing residue-wise fluctuations of the protein backbone. C: Radius of gyration (Rg) profile showing the compactness of the protein structure. D: Solvent-accessible surface area (SASA) illustrating changes in surface exposure. E: Number of hydrogen bonds formed between the ligand and protein over the simulation time. The data indicate moderate ligand flexibility and intermittent hydrogen bonding, with the protein maintaining overall structural stability.

The protein RMSD in both systems remained within a stable range, fluctuating between 0.5–3.5 Å for the MolPort-002-705-878 complex and 1.0–3.25 Å for the MolPort-007-550-904 complex, indicating overall structural stability of the receptor (Figs 6A and 7A). Among the leads, MolPort-002-705-878 exhibited an RMSD range of 0.25–2.0 Å, with an initial rapid increase to ∼2 Å followed by stabilization within a narrow 1.5–2.0 Å band, consistent with a rigid and tightly bound conformation. In contrast, MolPort-007-550-904, while showing a similar overall RMSD range, exhibited greater fluctuation, gradually decreasing after an early peak and spending most of the simulation between 0.25–1.25 Å, suggesting relatively enhanced conformational flexibility.

RMSF (Root mean square fluctuation) analyses (Figs 6B and 7B) showed that while the majority of residues fluctuated below 2.0 Å, certain regions in the MolPort-002-705-878 complex exhibited fluctuations reaching up to 6.0 Å, indicating localized flexibility. The radius of gyration (Rg) remained consistent in both simulations, with values fluctuating narrowly around 21.0–21.7 Å (Figs 6C and 7C), suggesting maintenance of a compact protein fold.

SASA (Solvent accessible surface area) trends (Figs 6D and 7D) indicated a slight decrease for the MolPort-007-550-904 complex, whereas MolPort-002-705-878 showed a modest increase, reflecting minor structural adjustments. Hydrogen bond analysis (Figs 6E and 7E) revealed that the MolPort-007-550-904 complex formed 1–2 transient hydrogen bonds on average, while the MolPort-002-705-878 complex consistently maintained 3–4 bonds, occasionally peaking at 6, implying a more stable and potentially higher-affinity interaction within the ligand-binding site of the protein [20].

MolPort-002-705-878 forms a more stable and persistent complex with 6JQR, as evidenced by lower ligand RMSD, stronger hydrogen bonding, and increased solvent exposure, whereas MolPort-007-550-904 exhibits greater conformational mobility and weaker stabilizing interactions, indicating a more transient binding mode [56].

### MM/GBSA binding free energy

**Fig 8:**
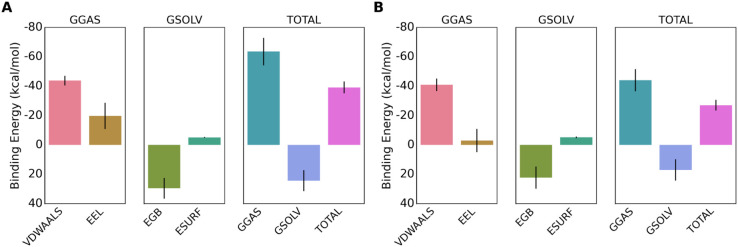
Illustration of MM/GBSA binding energy component analysis. A: MolPort-002-705-878. B: MolPort-007-550-904.

The van der Waals (VDWAALS) and electrostatic (EEL) contributions for MolPort-002-705-878 were –43.83 kcal/mol and –19.74 kcal/mol, respectively, while for MolPort-007-550-904 they were –41.01 kcal/mol and –3.08 kcal/mol. The polar solvation energy (EGB) was 29.48 kcal/mol for MolPort-002-705-878 and 22.24 kcal/mol for MolPort-007-550-904, and non-polar solvation (ESURF) contributions were –5.14 kcal/mol and –5.18 kcal/mol, respectively. Gas-phase interaction (GGAS) and solvation energies (GSOLV) were –63.57/24.34 kcal/mol for MolPort-002-705-878 and –44.08/17.05 kcal/mol for MolPort-007-550-904. Collectively, these components yielded binding free energies (ΔG) of –39.23 kcal/mol and –27.03 kcal/mol, confirming stronger overall binding for MolPort-002-705-878. The MM/GBSA energy profiles (Fig 8, Table 4) align with dynamic and structural stability observed in simulations, reinforcing the drug-like potential of both ligands [57].

**Table 4:** MM/GBSA binding energy components of the lead compounds, MolPort-002-705-878 and MolPort-007-550-904, in kcal/mol.

Energy Component	MolPort-002-705-878	MolPort-007-550-904
**VDWAALS**	–43.83	–41.01
**EEL**	–19.74	–3.08
**EGB**	29.48	22.24
**ESURF**	–5.14	–5.18
**GGAS**	–63.57	–44.08
**GSOLV**	24.34	17.05
**TOTAL ΔG**	**–39.23**	**–27.03**

### Principal component analysis (PCA)

#### Eigenvalues of the covariance matrix.

**Fig 9:**
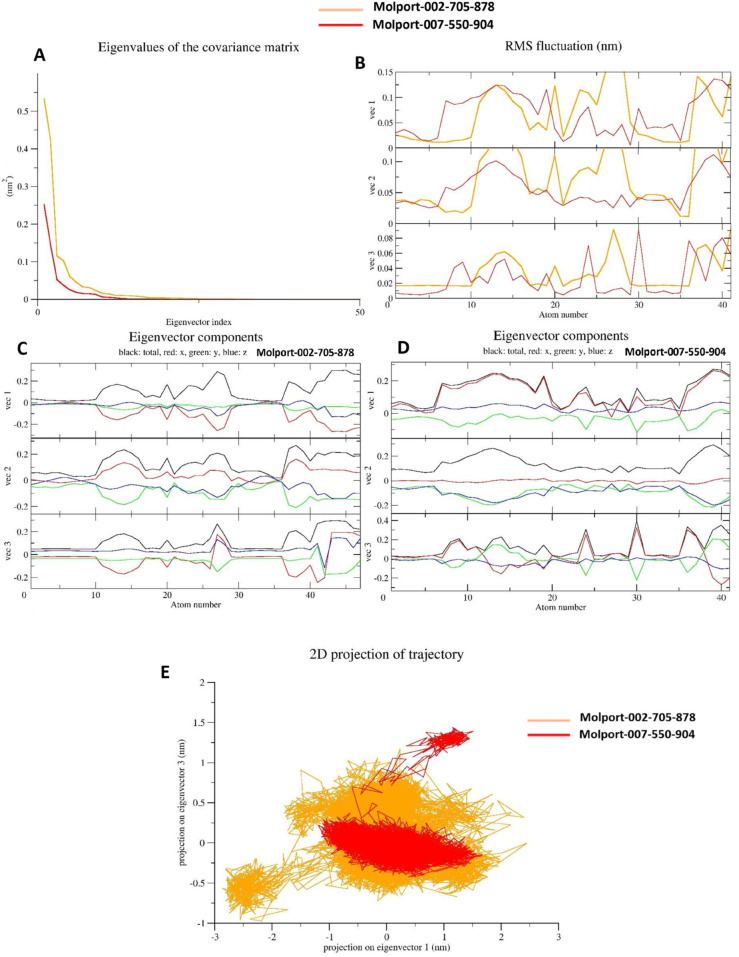
Principal component analysis (PCA) of the 6JQR–leads complexes. A: Eigenvalue distribution of the covariance matrix. B: RMS fluctuations along the first three principal eigenvectors. C: Eigenvector components (x, y, z) of the 6JQR–MolPort-002-705-878 complex. D: Eigenvector components (x, y, z) of the 6JQR–MolPort-007-550-904 complex. E: Two-dimensional projection of the trajectories along the first two principal components (vec1 vs. vec2). Overall, the PCA results indicate that MolPort-007-550-904 explores a broader conformational space with greater flexibility, whereas MolPort-002-705-878 exhibits more compact and stable dynamics.

The eigenvalue spectrum as shown in Panel A of Fig 9 illustrates the distribution of atomic fluctuations along the principal components. Both systems, 6JQR–(MolPort-002-705-878) complex (orange) and 6JQR–(MolPort-007-550-904) complex (red), exhibit a steep decline in the first few eigenvalues, followed by a long tail of lower-magnitude components. This trend indicates that the majority of the protein’s collective motions are captured within the first few principal components. The first complex displays slightly higher initial eigenvalues, suggesting stronger collective motions and potentially greater conformational variability compared to that of the latter complex.

#### RMS fluctuations along principal components.

Panel B of Fig 9 presents the root mean square (RMS) fluctuation of atomic positions along the first three principal eigenvectors (vec1, vec2, and vec3). The 6JQR–(MolPort-007-550-904) complex exhibits greater amplitude fluctuations, particularly along vec1 and vec3, with peaks exceeding 0.1 nm. These pronounced fluctuations imply more dynamic movements across key residues. In contrast, the 6JQR–(MolPort-002-705-878) complex demonstrates lower atomic fluctuations, indicative of a more rigid structure with constrained deviations along the principal axes [58].

#### Eigenvector component analysis.

Panels C (for the 6JQR–MolPort-002-705-878 complex) and D (for the 6JQR–MolPort-007-550-904 complex) of Fig 9 illustrate the x, y, and z components of the first three eigenvectors. In both systems, the total motion (black) is predominantly governed by the x-component (red), followed by moderate contributions from the y- (green) and z- (blue) directions. However, the MolPort-007-550-904 complex displays greater directional variation, especially in vec1 and vec2, indicating more spatially diverse and extended motion. The MolPort-002-705-878 complex shows more uniform directional behavior, suggesting a compact and directionally consistent dynamic profile [58].

#### 2D projection of trajectory.

Panel E of Fig 9 provides a two-dimensional projection of the atomic motions along the first two principal components (vec1 vs. vec2). The 6JQR–(MolPort-007-550-904) complex (in red) explores a wider and more dispersed conformational space, indicating the sampling of multiple structural states during the simulation. Conversely, the 6JQR–(MolPort-002-705-878) complex (in orange) remains tightly clustered within a narrow conformational region, reflecting greater structural rigidity and limited flexibility. This projection emphasizes the dynamic plasticity of the MolPort-007-550-904 complex relative to the more stable and conformationally constrained behavior of the MolPort-002-705-878 complex, which may correlate with differences in functional or binding characteristics [59].

### Free energy landscape (FEL)

**Fig 10:**
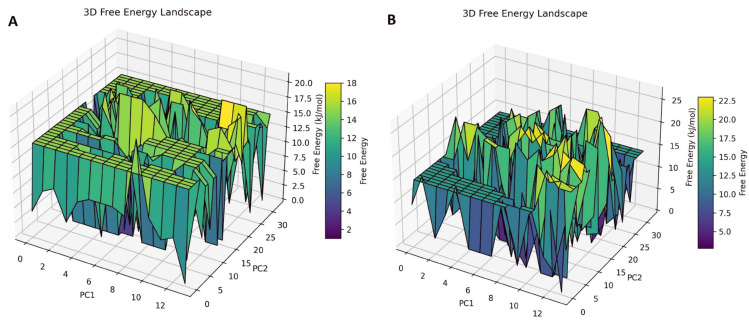
Free energy landscape (FEL) of the 6JQR–MolPort complexes. A: 6JQR–MolPort-007-550-904. B: 6JQR–MolPort-002-705-878.

FEL analysis of the 6JQR–(MolPort-007-550-904) complex (Panel A of Fig 10) exhibits a rugged topology characterized by multiple shallow and deep minima dispersed across the PC1–PC2 conformational space. This distribution indicates frequent transitions among diverse conformational states, suggesting a higher degree of structural flexibility and the presence of several semi-stable intermediates sampled throughout the simulation [60]. In contrast, the FEL of the 6JQR–(MolPort-002-705-878) complex (Panel B of Fig 10) presents a more compact yet energetically heterogeneous landscape. While several peaks and troughs are evident, the overall topology is more condensed, featuring fewer and narrower basins. The presence of deep energy wells suggests that the system adopts fewer, but more stable, conformational states compared to the MolPort-007-550-904 complex.

### Quantum properties evaluation

#### Geometry optimization.

**Fig 11:**
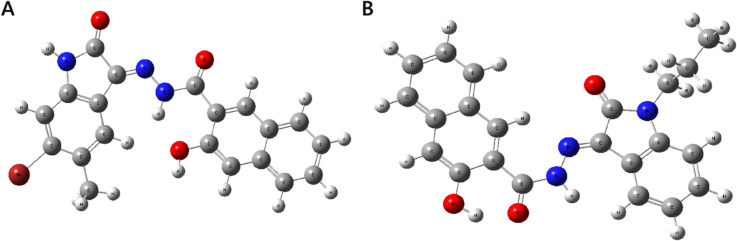
Optimized molecular geometries of the lead molecules. A: MolPort-002-705-878. B: MolPort-007-550-904.

Geometry optimization confirmed that both leads adopt geometrically stable conformations, as evidenced by their very low RMS Cartesian force values [61], with the optimized molecular structures shown in Fig 11 and key geometric and electronic parameters summarized in Table 5. MolPort-002-705-878 exhibits a significantly lower global minimum energy (–3734.930 Hartree) than MolPort-007-550-904 (–1240.047 Hartree), indicating greater thermodynamic stability [61]. Its dipole moment (8.027 Debye) also exceeds that of MolPort-007-550-904 (6.411 Debye), indicating a stronger potential for polar interactions within the FLT3 binding site [62].

**Table 5:** Calculated electronic energy, dipole moment, and RMS cartesian force of the lead compounds.

Compounds	Dipole Moment (*μ*) (Debye)	Electronic Energy (Hartree)	RMS Cartesian Force (Hartree/Bohr)
**MolPort-002-705-878**	8.027	–3734.930	0.000006
**MolPort-007-550-904**	6.411	–1240.047	0.000003

#### Frontier molecular orbitals (FMOs).

**Fig 12:**
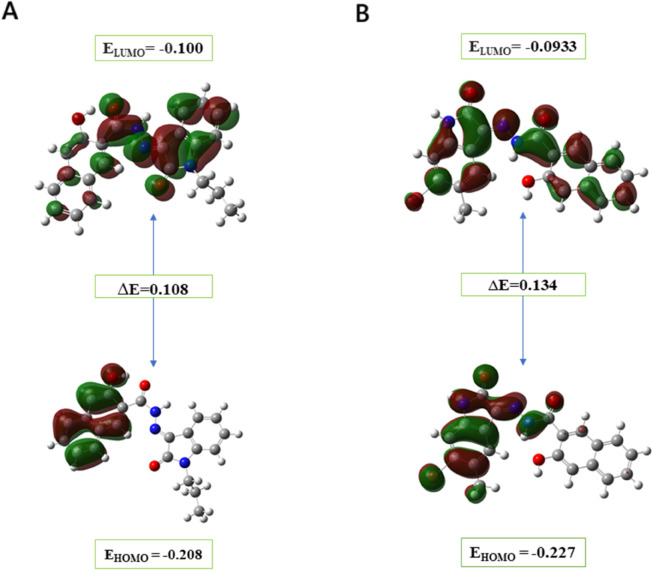
Frontier molecular orbitals (HOMO and LUMO) at the ground state for leads. A: MolPort-002-705-878. B: MolPort-007-550-904. The smaller HOMO–LUMO gap of MolPort-007-550-904 indicates greater chemical softness and higher reactivity relative to the next lead.

Frontier molecular orbitals (FMOs) provide qualitative insight into the likelihood of electron transfer between the HOMO (highest occupied molecular orbital) and LUMO (lowest unoccupied molecular orbital), with their spatial distributions illustrated in Fig 12. MolPort-007-550-904 shows a higher HOMO energy (–0.208 eV) and lower LUMO energy (–0.100 eV) than MolPort-002-705-878 (HOMO: –0.227 eV, LUMO: –0.0933 eV), indicating distinct electron-donating and -accepting tendencies. The HOMO–LUMO energy gaps for MolPort-007-550-904 and MolPort-002-705-878 are calculated to be 0.108 eV and 0.134 eV, respectively, with the smaller gap of MolPort-007-550-904 indicating greater chemical softness and higher reactivity, thereby facilitating more efficient electron transfer [63].

#### Global reactivity parameters.

Global reactivity parameters further distinguish the two leads, as shown in Table 6. The greater softness of MolPort-007-550-904 (18.518) suggests higher polarizability and reactivity compared to MolPort-002-705-878 (14.970). Their low chemical potentials (MolPort-007-550-904: –0.154 eV; MolPort-002-705-878: –0.160 eV) further support electron-donating behavior in biological environments [64]. Both exhibit positive chemical hardness and electrophilicity index values, indicating potential to modulate protein–ligand binding and participate in charge transfer [65], while the higher electronegativity and hardness of MolPort-002-705-878 suggest greater electronic stability and lower susceptibility to perturbation compared to MolPort-007-550-904 [66]. Collectively, these descriptors imply that while both molecules are chemically active, MolPort-007-550-904 may exhibit stronger interaction potential through more facile electron transfer.

**Table 6:** Computed global reactivity descriptors (in eV) for two leads.

Parameter	MolPort-002-705-878	MolPort-007-550-904
**Ionization energy (I)**	0.227	0.208
**Electron affinity (A)**	0.093	0.100
**Chemical hardness (*η*)**	0.069	0.054
**Chemical softness (*σ*)**	14.970	18.518
**Electronegativity (χ)**	0.160	0.154
**Chemical potential (*μ*)**	–0.160	–0.154
**Electrophilicity (*ω*)**	0.192	0.219

#### Molecular electrostatic potential (MEP).

**Fig 13:**
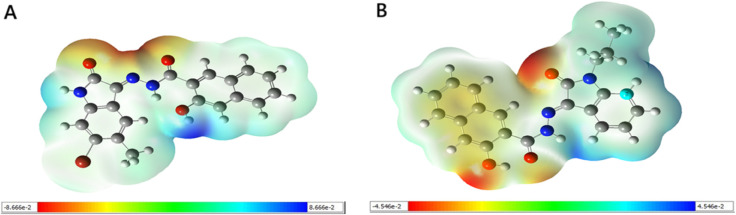
Molecular electrostatic potential (MEP) maps of leads. A: MolPort-002-705-878. B: MolPort-007-550-904. The color-coded isodensity surfaces highlight regions of electron-rich (red, nucleophilic) and electron-poor (blue, electrophilic) potential, providing insight into possible reactive sites and intermolecular interactions.

The color-mapped molecular electrostatic potential (MEP) surface visualizes local charge distribution, with red indicating electron-rich regions favorable for electrophilic attack and blue marking electron-deficient sites prone to nucleophilic interaction [67]. Green denotes areas of near-zero potential, while intermediate colors (orange, yellow, green) represent the gradient between extremes. As depicted in Fig 13, both leads exhibit negative electrostatic potential localized around electronegative atoms such as nitrogen and oxygen, indicating prominent nucleophilic sites. In contrast, positive potential appears near hydrogen atoms, suggesting electrophilic character, while carbon atoms display mixed behavior depending on their local chemical context. These regions, prone to nucleophilic and electrophilic attacks, inform possible interaction modes with biological macromolecules by complementing the electrostatic landscape of the protein binding pocket [68].

#### Natural bond orbital analysis (NBO).

NBO analysis revealed significant intramolecular charge transfer (ICT) interactions in both lead compounds, contributing to electronic stability (S2 Table and S3 Table). In MolPort-002-705-878, the dominant donor–acceptor interaction was LP(1)N6 → $\pi^{*}$(C3–O19), with a stabilization energy of 48.74 kcal/mol, alongside other notable interactions; LP(1)N13 → $\pi^{*}$(C8–O20), LP(1)N6 → $\pi^{*}$(C4–C12), and LP(2)O19 → $\sigma^{*}$(C3–N6), ranging from 28.74 to 46.92 kcal/mol. In MolPort-007-550-904, a strong LP(1)C14 → $\pi^{*}$(C5–C9) interaction was observed, with an *E*(2) value of 84.40 kcal/mol, alongside additional stabilizing transitions from LP(1)N3 and LP(1)N11 into conjugated $\pi^{*}$ orbitals. High second-order perturbation energies (*E*(2)) indicate significant lone pair to $\pi^{*}$ and $\sigma^{*}$ delocalization and resonance stabilization, particularly in heteroatom-rich regions, contributing to molecular rigidity and thermodynamic stability [51,68]. These conjugation patterns may enhance pharmacophoric alignment and binding complementarity with the FLT3 active site through hydrogen bonding, *π*–*π* stacking, and electrostatic interactions [69].

#### Mulliken and natural population analyses.

**Fig 14:**
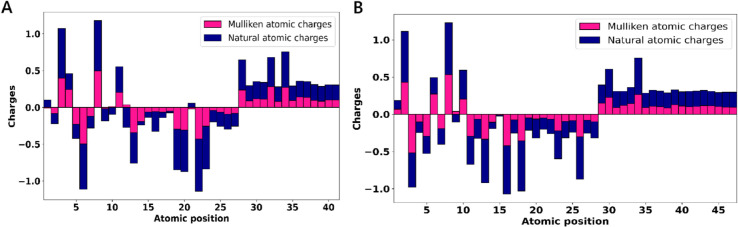
Illustrations of Mulliken and natural atomic charges of the leads. A: MolPort-002-705-878. B: MolPort-007-550-904. Positive and negative charges are plotted in diverging directions from zero, with stacked bars for same-signed contributions. These charge distributions reveal differences in electron localization and polarity, highlighting potential reactive centers within each molecule.

Mulliken and natural population charge analyses are widely used to identify reactive sites, model electrostatic interactions, and support force field parameterization in molecular simulations [70]. In both leads, C8 was identified as the most electron-deficient atom, while the most electron-rich centers varied according to Mulliken and NBO-based natural charge analyses: N3 and O18 in MolPort-007-550-904, and N6 and O22 in MolPort-002-705-878 (Fig 14 and S4 Table), reflecting substantial charge localization at key electronegative centers relevant for binding [71]. These sites were also involved in interactions during docking analysis, consistent with charge-based predictions.

The bromine atom (Br21) in MolPort-002-705-878 carried a small positive Mulliken and natural charge, suggesting reduced electronic reactivity compared to typical halogen atoms, likely due to electron delocalization or shielding by adjacent functional groups [72]. Among hydrogen atoms, H34 in MolPort-007-550-904 and H32 in MolPort-002-705-878 exhibited the highest Mulliken charges, suggesting potential roles in electrostatic or hydrogen bonding interactions [73], while carbon atoms displayed both positive and negative charges reflecting their diverse electronic environments.

### ADMET analysis

The pharmacokinetic and toxicological characteristics of the lead compounds were evaluated through comprehensive *in silico* analyses to assess their potential as drug-like molecules suitable for preclinical development. MolPort-002-705-878 exhibits a favorable absorption profile, with high predicted gastrointestinal (GI) permeability and compliance with key criteria for drug-likeness, as set by Lipinski, Ghose, Veber, and Egan rules. The compound exhibits moderate lipophilicity (consensus LogP = 3.47), a topological polar surface area (TPSA) of 90.79 Å^2^, and contains three rotatable bonds, which collectively support passive diffusion across biological membranes [25,74]. Despite being moderately soluble by ESOL and poorly soluble by AliLogS and Silicos-IT models, its drug-likeness is reinforced by favorable bioavailability (score: 0.55) and minimal synthetic complexity. Importantly, the compound is predicted to be a non-substrate for P-glycoprotein and lacks blood–brain barrier permeability (S4 Fig). The predicted Lethal Dose 50 (LD_50_) for this lead is approximately 3009 mg/kg, corresponding to toxicity class V under the Globally Harmonized System (GHS), indicative of low acute toxicity [75]. It is estimated to be non-carcinogenic, non-mutagenic, and non-cytotoxic. Organ-specific toxicity predictions suggest a low likelihood of cardiotoxicity and respiratory toxicity, whereas potential hepatotoxicity, nephrotoxicity, and neurotoxicity merit careful consideration in future experimental studies. Additionally, molecular initiating event (MIE) analyses further indicate activation of the aryl hydrocarbon receptor (AhR) and potential mitochondrial membrane disruption, both of which may warrant further investigation during safety profiling.

MolPort-007-550-904, similarly, demonstrates high predicted GI absorption and complies with all major drug-likeness rules, aside from a single Muegge violation. It shows slightly higher structural flexibility, with five rotatable bonds and a TPSA of 82.00 Å^2^. Although its consensus LogP (3.46) and solubility predictions mirror the first compound, its slightly elevated fraction of sp^3^ carbons may suggest better metabolic stability [76]. As with MolPort-002-705-878, it is not BBB-permeant and avoids P-glycoprotein-mediated efflux (S4 Fig). However, it displays a broader cytochrome P450 (CYP) inhibition profile *in silico*, inhibiting CYP1A2, CYP2C19, CYP2C9, and CYP3A4, raising concerns regarding metabolic interactions and potential drug–drug interactions [77]. Given these findings, experimental validation through cytotoxicity assays and metabolic interaction studies is essential to assess clinical relevance and mitigate potential risks during development. In toxicity evaluation, this lead has comparatively lower LD_50_ of 1400 mg/kg and it is classified as toxicity class IV, suggesting moderate acute toxicity [75]. While it exhibits low predicted probability of causing carcinogenicity, immunotoxicity, or endocrine disruption, it demonstrates broader toxicity liabilities, with predicted neurotoxicity, nephrotoxicity, respiratory toxicity, and mutagenicity. Although activation of nuclear receptor pathways appears minimal, this compound also engages in mitochondrial toxicity mechanisms. Taken together, these findings underscore the need for comprehensive *in vitro* safety profiling to confirm computational estimates and clarify the clinical relevance of observed liabilities.

## Conclusion

This investigation focuses on FLT3, aiming to discover innovative small molecules with significant therapeutic potential. In this study, the structure-based screening tool Pharmit and the comprehensive commercial chemical library MolPort were leveraged to maximize chemical diversity and increase the likelihood of identifying highly potent and selective FLT3 inhibitors. The rigorous process began with an initial virtual screening using Pharmit, followed by docking-based screening of 419 selected hits, resulting in the identification of two promising lead molecules: MolPort-002-705-878 and MolPort-007-550-904. Additional validation of these leads was performed using molecular dynamics simulations to probe the stability of protein–ligand binding, density functional theory (DFT) exploration to characterize quantum chemical behavior, and ADMET profiling to evaluate drug-likeness, all of which collectively support these leads as potential FLT3 antagonists. Importantly, these compounds occupy chemical space distinct from FDA-approved FLT3 inhibitors, particularly in their scaffold architecture, which features rigid benzolactam–amide frameworks and a characteristic o-HNCA motif, replacing the bulky, nitrogen-rich polycyclic cores of existing drugs. This structural novelty underscores their potential for differentiated binding profiles and supports their prioritization for further pharmacological assessment. However, it must be clearly stated that these are computationally derived leads. While the multi-tier validation provides strong preliminary evidence, biological testing is essential to confirm their activity, specificity, and safety before they can be considered viable preclinical candidates. This study exemplifies a scalable, data-driven framework for next-generation drug discovery with broad applicability to other biomolecular targets across diverse disease areas. Nevertheless, key limitations remain, including high computational demands, reliance on *in silico* predictions without experimental confirmation, and the potential incompleteness of commercial chemical libraries. Addressing these challenges will require future efforts to integrate high-performance computing infrastructure, strengthen collaboration with experimental pharmacology, and incorporate emerging non-commercial compound repositories. As the field evolves, harmonizing computational insights with experimental rigor will be essential for transforming virtual hits into clinically meaningful candidates.

## Supporting information

S1 Fig Generated pharmacophores in Gliteritinib within FLT3 protein. Two white zones represent hydrogen donor region, orange represents hydrogen-bond acceptor, while green represents hydrophobic region.
(TIFF)

S2 Fig Structural comparison of lead compounds with FDA-approved FLT3 inhibitors. A: Gilteritinib. B: Midostaurin. C: Quizartinib. FDA-approved inhibitors (A–C) feature bulky, heteroaromatic scaffolds with nitrogen-rich and/or fluorinated motifs. D: MolPort-002-705-878. E: MolPort-007-550-904. The identified leads (D–E) share a compact benzolactam–amide framework and an ortho-hydroxynaphthalene-1-carboxamide (o-HNCA) motif, which is absent in current therapeutics.
(TIFF)

S3 Fig Pairwise Tanimoto similarity among lead compounds and FDA-approved FLT3 inhibitors. Similarity was calculated using ECFP4 fingerprints (2048 bits). Both MolPort-002-705-878 and MolPort-007-550-904 exhibit low 2D similarity (Tanimoto coefficient < 0.20) with Gilteritinib, Midostaurin, and Quizartinib, underscoring their scaffold-level novelty.
(TIFF)

S4 Fig BOILED-Egg model generated by SwissADME for MolPort-002-705-878 and MolPort-007-550-904. The model illustrates predicted blood–brain barrier (BBB) permeability and P-glycoprotein (P-gp) efflux status.
(TIFF)

S1 Table MolPort Compound IDs selected for docking after pharmacophore filtering. (PDF)

S2 Table Second-order perturbation analysis of the interaction between donor and acceptor orbitals of compound MolPort-002-705-878 in the NBO basis. (PDF)

S3 Table Second-order perturbation analysis of the interaction between donor and acceptor orbitals of compound MolPort-007-550-904 in the NBO basis. (PDF)

S4 Table Mulliken and natural charges of all the atoms in MolPort-007-550-904 and MolPort-002-705-878 molecules. (PDF)
