# Supplementary figures and images for: Structure-guided discovery and characterization of novel FLT3 inhibitors for acute myeloid leukemia treatment
Source: PLoS One. 2025 Oct 13;20(10):e0334415. doi: 10.1371/journal.pone.0334415 (PMC12517515; doi:10.1371/journal.pone.0334415)

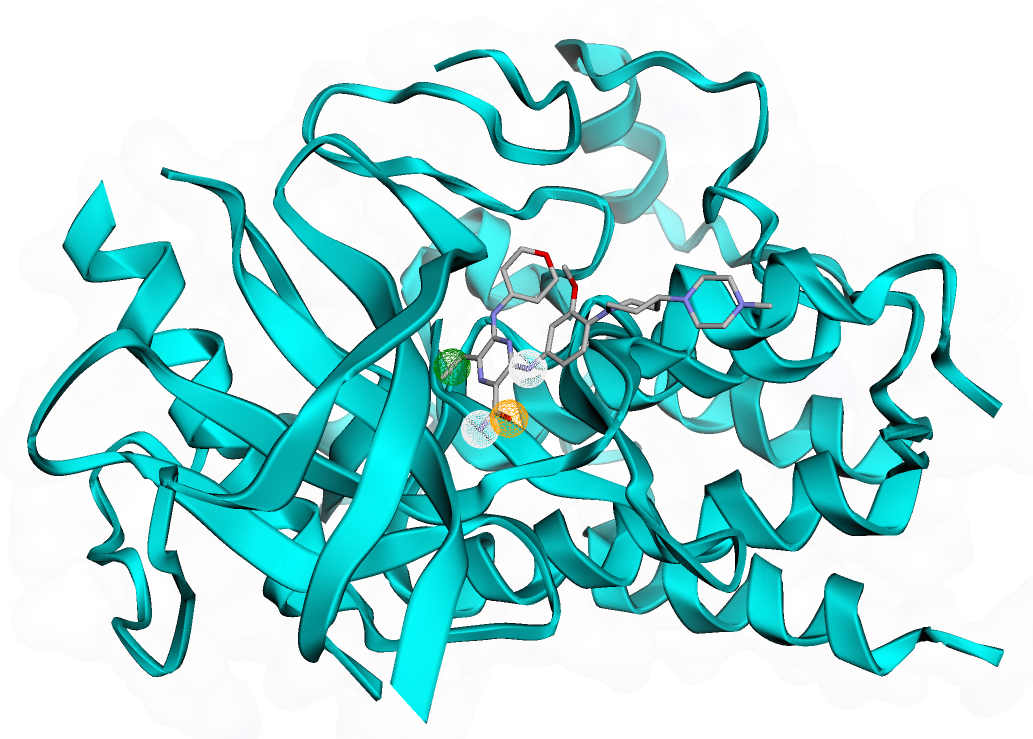

Supplement: S1 Fig — Two white zones represent hydrogen donor region, orange represents hydrogen-bond acceptor, while green represents hydrophobic region. (TIFF) [file pone.0334415.s001.tif]

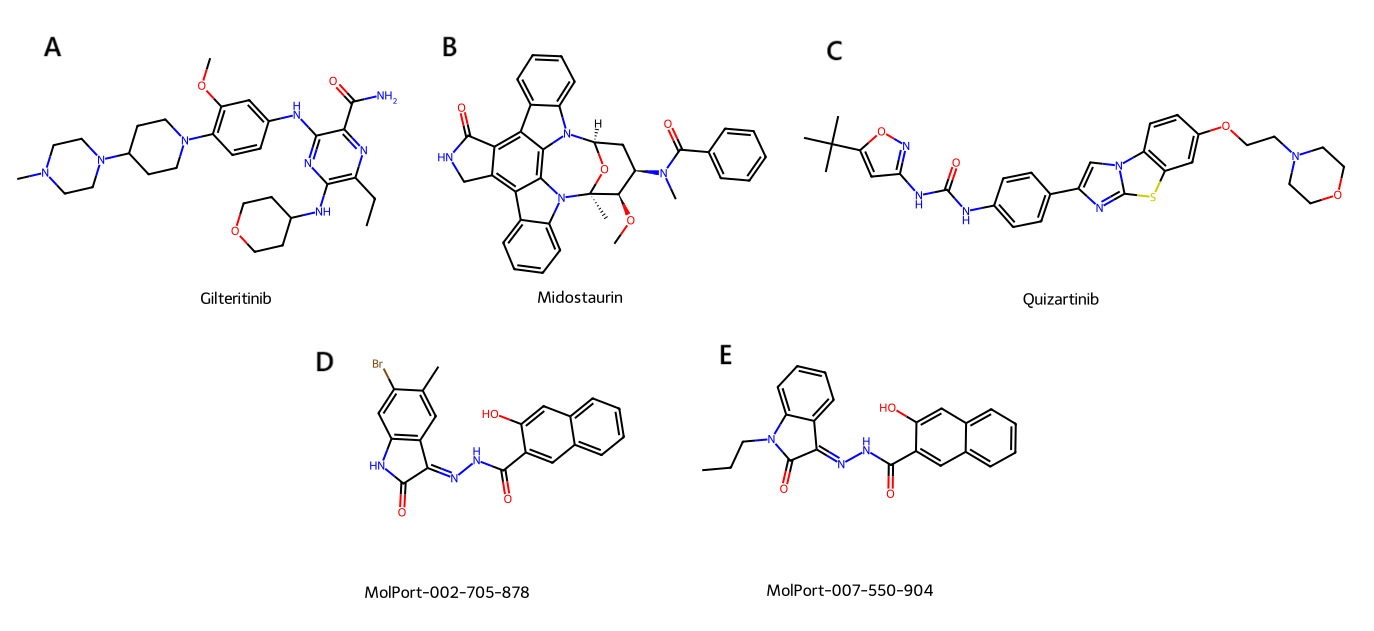

Supplement: S2 Fig — A: Gilteritinib. B: Midostaurin. C: Quizartinib. FDA-approved inhibitors (A–C) feature bulky, heteroaromatic scaffolds with nitrogen-rich and/or fluorinated motifs. D: MolPort-002-705-878. E: MolPort-007-550-904. The identified leads (D–E) share a compact benzolactam–amide framework and an ortho-hydroxynaphthalene-1-carboxamide (o-HNCA) motif, which is absent in current therapeutics. (TIFF) [file pone.0334415.s002.tif]

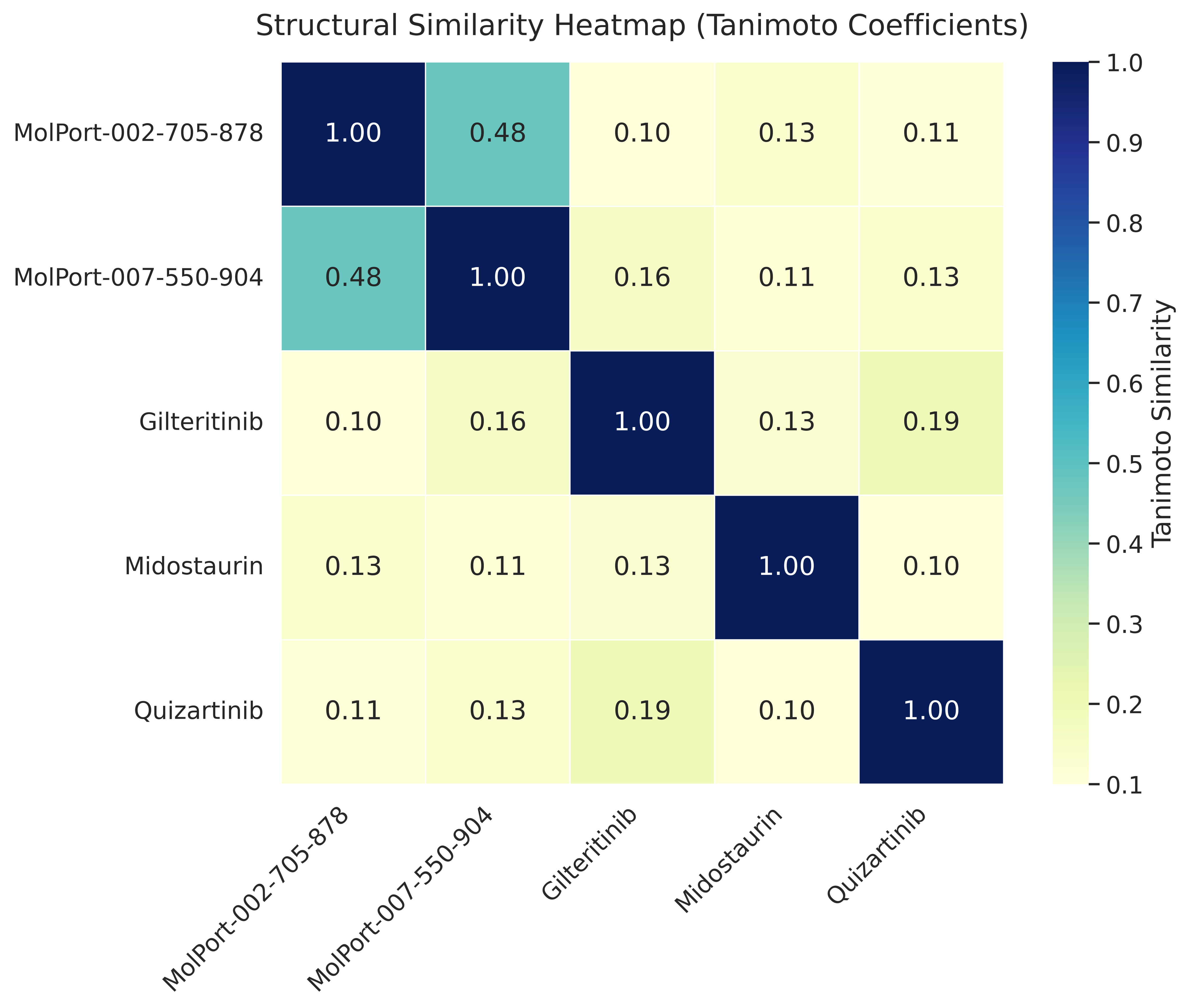

Supplement: S3 Fig — Similarity was calculated using ECFP4 fingerprints (2048 bits). Both MolPort-002-705-878 and MolPort-007-550-904 exhibit low 2D similarity (Tanimoto coefficient < 0.20) with Gilteritinib, Midostaurin, and Quizartinib, underscoring their scaffold-level novelty. (TIFF) [file pone.0334415.s003.tif]

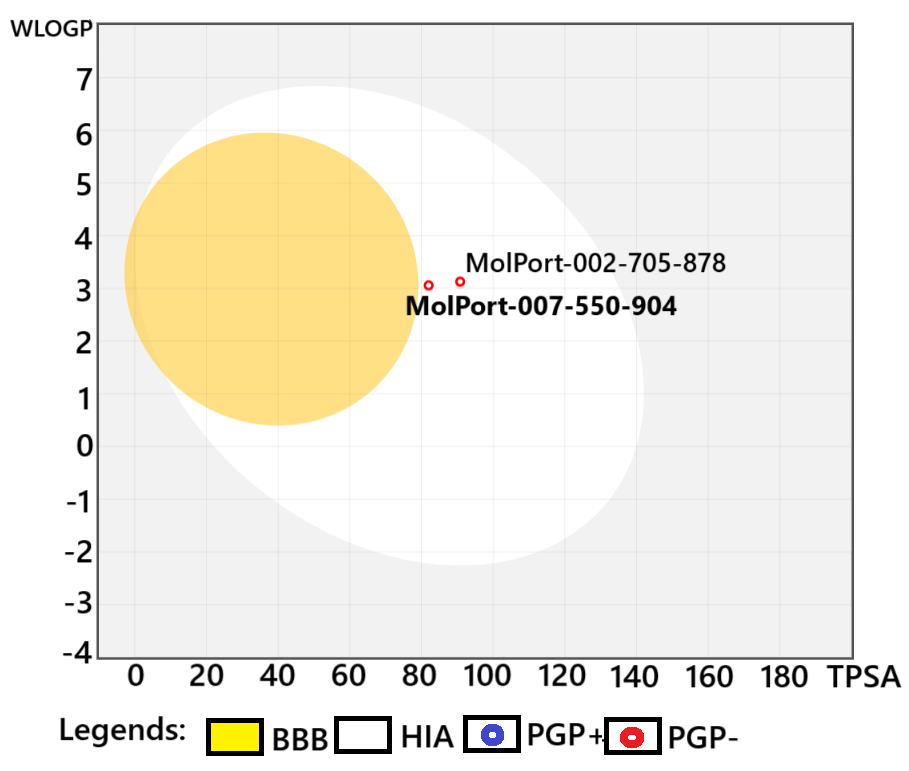

Supplement: S4 Fig — The model illustrates predicted blood–brain barrier (BBB) permeability and P-glycoprotein (P-gp) efflux status. (TIFF) [file pone.0334415.s004.tif]
