# Supplementary material for: Structure-guided discovery and characterization of novel FLT3 inhibitors for acute myeloid leukemia treatment
Source: PLoS One. 2025 Oct 13;20(10):e0334415. doi: 10.1371/journal.pone.0334415 (PMC12517515; doi:10.1371/journal.pone.0334415)
Supplement: S1 Table — (PDF) [file pone.0334415.s005.pdf]

S1 Table: MolPort Compound IDs selected for docking after pharmacophore filtering.

Compounds	Compounds	Compounds	Compounds
MolPort-002-705-878	MolPort-046-857-239	MolPort-001-028-598	MolPort-002-655-342
MolPort-007-903-602	MolPort-002-329-300	MolPort-000-732-736	MolPort-019-911-361
MolPort-019-910-904	MolPort-003-714-345	MolPort-002-945-344	MolPort-006-844-396
MolPort-007-550-904	MolPort-001-529-307	MolPort-002-664-858	MolPort-019-707-345
MolPort-007-606-024	MolPort-001-544-997	MolPort-019-911-366	MolPort-002-037-067
MolPort-004-856-891	MolPort-001-539-548	MolPort-008-288-055	MolPort-002-629-529
MolPort-001-650-645	MolPort-004-856-888	MolPort-008-286-984	MolPort-002-664-450
MolPort-001-671-202	MolPort-006-804-052	MolPort-002-486-321	MolPort-004-850-504
MolPort-000-817-743	MolPort-001-521-829	MolPort-001-495-409	MolPort-002-666-266
MolPort-004-856-885	MolPort-000-225-262	MolPort-001-539-207	MolPort-002-668-549
MolPort-004-846-897	MolPort-004-942-287	MolPort-047-920-301	MolPort-002-563-446
MolPort-047-019-496	MolPort-001-610-192	MolPort-046-703-376	MolPort-018-580-482
MolPort-001-647-167	MolPort-046-689-686	MolPort-002-672-426	MolPort-002-667-910
Molport-051-903-815	MolPort-002-668-584	MolPort-000-431-547	MolPort-001-645-325
MolPort-051-700-067	MolPort-002-665-607	MolPort-002-667-942	MolPort-002-668-107
MolPort-002-664-137	MolPort-002-671-622	MolPort-001-644-819	MolPort-051-755-753
MolPort-002-938-003	MolPort-002-046-193	MolPort-001-549-987	Molport-051-838-512
MolPort-000-851-496	MolPort-000-858-348	MolPort-002-989-879	MolPort-002-665-252
MolPort-001-620-913	MolPort-002-664-182	MolPort-002-251-242	MolPort-002-134-370
MolPort-007-987-586	MolPort-002-667-049	MolPort-044-435-979	MolPort-002-667-454
MolPort-002-566-143	MolPort-004-878-609	MolPort-001-981-118	MolPort-002-817-646
MolPort-001-639-043	MolPort-001-639-878	MolPort-002-212-894	MolPort-000-884-203
MolPort-007-662-228	MolPort-002-139-081	MolPort-001-954-874	MolPort-002-134-368
MolPort-000-225-285	MolPort-018-542-219	MolPort-035-946-294	MolPort-003-143-942
MolPort-004-850-680	MolPort-002-838-441	MolPort-051-700-066	MolPort-002-212-495
MolPort-047-355-235	MolPort-002-139-019	MolPort-009-652-536	MolPort-002-539-711
MolPort-002-507-745	MolPort-002-563-305	MolPort-002-665-369	MolPort-002-666-245
MolPort-002-663-032	MolPort-002-668-772	MolPort-002-564-370	Molport-051-892-177
MolPort-002-247-103	MolPort-002-607-129	MolPort-002-563-677	MolPort-002-153-986
MolPort-002-735-434	MolPort-039-050-507	MolPort-002-251-118	MolPort-001-912-640
MolPort-002-469-922	MolPort-002-666-462	MolPort-002-666-949	MolPort-002-134-369
MolPort-049-225-129	MolPort-002-668-414	MolPort-004-850-679	MolPort-038-402-714
MolPort-000-859-344	MolPort-001-649-761	MolPort-001-539-825	MolPort-023-332-859
MolPort-019-913-459	MolPort-003-846-724	MolPort-002-507-746	MolPort-000-765-456
MolPort-000-555-518	MolPort-002-470-753	MolPort-002-668-265	MolPort-004-933-308
MolPort-002-507-747	MolPort-002-547-197	MolPort-002-599-502	MolPort-001-544-405
MolPort-000-650-811	MolPort-002-468-571	MolPort-001-586-477	MolPort-002-566-100

Compounds	Compounds	Compounds	Compounds
MolPort-002-602-189	MolPort-001-893-495	MolPort-002-667-070	MolPort-002-668-973
MolPort-002-665-240	MolPort-002-139-031	MolPort-002-902-191	MolPort-001-490-415
MolPort-001-620-497	MolPort-047-156-478	MolPort-002-640-643	MolPort-045-967-963
MolPort-002-566-538	MolPort-002-003-681	MolPort-002-247-093	MolPort-002-635-812
MolPort-000-934-045	MolPort-003-802-422	MolPort-002-565-489	MolPort-047-933-048
MolPort-019-797-234	MolPort-002-585-457	MolPort-002-602-969	MolPort-002-667-991
MolPort-002-745-815	MolPort-002-601-356	MolPort-002-664-939	MolPort-002-567-705
MolPort-002-604-045	MolPort-002-673-970	MolPort-002-816-077	MolPort-002-603-395
MolPort-000-921-623	MolPort-003-355-489	MolPort-004-788-067	MolPort-002-345-146
MolPort-047-586-059	MolPort-027-352-440	MolPort-002-667-688	Molport-004-802-782
Molport-051-901-940	MolPort-002-587-437	MolPort-002-507-743	MolPort-049-220-517
MolPort-001-014-891	MolPort-002-003-758	MolPort-002-600-113	MolPort-002-565-702
MolPort-009-753-114	MolPort-001-610-110	MolPort-002-568-090	MolPort-000-919-403
MolPort-002-605-666	MolPort-047-798-457	MolPort-003-875-129	MolPort-002-136-586
MolPort-035-689-865	MolPort-002-600-911	MolPort-000-851-136	MolPort-004-262-677
MolPort-002-048-223	MolPort-000-224-828	Molport-051-810-669	MolPort-004-942-293
MolPort-002-602-566	MolPort-002-250-837	MolPort-002-663-314	MolPort-001-955-424
MolPort-002-121-860	MolPort-035-763-685	MolPort-002-705-687	MolPort-046-510-951
MolPort-002-604-343	MolPort-048-722-691	MolPort-004-345-557	MolPort-000-801-443
MolPort-004-350-349	MolPort-002-798-643	Molport-004-802-783	MolPort-002-563-493
MolPort-002-568-543	MolPort-002-173-815	MolPort-046-791-775	MolPort-028-914-300
MolPort-002-635-848	MolPort-035-685-038	MolPort-010-466-524	MolPort-002-251-549
MolPort-000-678-736	MolPort-002-625-922	MolPort-015-143-278	MolPort-022-375-119
MolPort-000-734-890	MolPort-028-957-496	MolPort-051-765-205	MolPort-035-689-869
Molport-051-804-083	MolPort-029-940-689	MolPort-002-666-483	MolPort-000-482-961
MolPort-028-750-277	MolPort-000-149-495	MolPort-003-818-711	MolPort-039-322-891
MolPort-007-899-726	MolPort-004-330-336	MolPort-047-970-305	MolPort-046-528-459
MolPort-016-625-795	MolPort-047-970-407	MolPort-000-145-535	MolPort-002-816-887
MolPort-000-521-655	MolPort-002-748-021	MolPort-046-934-151	MolPort-009-196-316
MolPort-002-603-739	MolPort-001-661-776	MolPort-044-553-661	MolPort-002-563-576
MolPort-023-330-468	MolPort-014-880-695	MolPort-042-646-687	MolPort-049-242-540
MolPort-002-858-741	MolPort-044-832-228	MolPort-000-931-718	MolPort-046-194-590
Molport-051-804-543	Molport-045-019-282	MolPort-002-566-888	MolPort-047-967-835
MolPort-047-967-884	MolPort-047-740-535	MolPort-001-772-772	MolPort-002-319-269
MolPort-035-785-556	MolPort-002-462-096	MolPort-035-874-381	MolPort-047-969-126
MolPort-035-394-359	MolPort-000-146-043	MolPort-004-363-487	MolPort-013-209-953
MolPort-004-293-855	MolPort-000-885-524	MolPort-002-461-971	MolPort-039-332-433
MolPort-000-271-683	MolPort-001-899-637	MolPort-047-970-273	MolPort-027-679-828
MolPort-000-149-357	MolPort-002-471-423	MolPort-000-163-937	MolPort-051-766-167

Compounds	Compounds	Compounds	Compounds
MolPort-002-889-632	MolPort-046-940-595	MolPort-002-563-218	MolPort-019-930-873
MolPort-046-522-317	MolPort-003-920-584	MolPort-002-041-254	MolPort-004-955-729
MolPort-038-438-241	MolPort-006-716-553	MolPort-022-379-265	MolPort-001-790-840
MolPort-002-462-058	MolPort-002-462-059	MolPort-002-628-504	MolPort-027-948-434
MolPort-001-761-481	Molport-051-912-158	MolPort-000-147-051	MolPort-046-934-207
MolPort-001-771-862	MolPort-002-566-786	MolPort-001-649-791	MolPort-022-898-151
MolPort-046-860-003	MolPort-049-202-162	MolPort-002-568-945	MolPort-051-649-859
MolPort-015-141-656	MolPort-039-203-876	MolPort-038-434-884	MolPort-003-904-445
MolPort-004-363-488	MolPort-047-413-669	MolPort-051-544-861	MolPort-000-704-473
MolPort-006-716-291	MolPort-044-183-544	MolPort-004-291-541	MolPort-023-330-328
MolPort-002-041-266	MolPort-000-271-686	MolPort-023-330-096	MolPort-044-817-891
MolPort-044-558-710	MolPort-003-749-417	MolPort-000-271-687	MolPort-042-681-224
MolPort-047-970-797	MolPort-039-203-878	MolPort-004-767-950	MolPort-019-878-674
MolPort-009-196-794	MolPort-016-578-683	MolPort-038-386-980	MolPort-038-521-591
MolPort-027-948-360	MolPort-009-199-618	MolPort-003-929-057	MolPort-046-686-088
MolPort-035-690-033	MolPort-001-814-813	MolPort-005-311-290	MolPort-003-847-744
MolPort-051-589-588	MolPort-042-672-242	MolPort-009-199-188	MolPort-051-767-274
MolPort-020-248-041	MolPort-047-413-648	MolPort-000-210-149	MolPort-011-016-227
MolPort-047-413-628	MolPort-023-332-377	MolPort-016-582-338	MolPort-016-579-028
MolPort-004-767-393	MolPort-006-710-144	MolPort-027-947-944	MolPort-035-687-287
MolPort-004-768-362	MolPort-004-949-903	MolPort-023-222-588	MolPort-023-331-591
MolPort-004-812-210	MolPort-044-557-618	MolPort-002-719-266	MolPort-046-196-178
Molport-051-891-697	MolPort-019-879-186	MolPort-020-222-334	MolPort-000-210-144
MolPort-000-146-587	MolPort-047-352-420	MolPort-004-816-169	MolPort-020-217-041
MolPort-008-000-002	MolPort-046-194-946	MolPort-004-768-381	MolPort-001-893-025
MolPort-016-583-176	MolPort-019-996-089	MolPort-004-767-492	MolPort-000-884-453
MolPort-008-646-580	MolPort-020-003-025	MolPort-047-598-283	MolPort-002-121-685
MolPort-000-720-481	MolPort-046-424-804	MolPort-000-841-538	MolPort-022-628-246
MolPort-039-137-277	MolPort-004-942-582	MolPort-004-891-628	
