# Supplementary material for: Structure-guided discovery and characterization of novel FLT3 inhibitors for acute myeloid leukemia treatment
Source: PLoS One. 2025 Oct 13;20(10):e0334415. doi: 10.1371/journal.pone.0334415 (PMC12517515; doi:10.1371/journal.pone.0334415)
Supplement: S2 Table — (PDF) [file pone.0334415.s006.pdf]

S2 Table: Second-order perturbation analysis of the interaction between donor and acceptor orbitals of compound MOLPORT-002-705-878 in NBO basis.

Donor NBO(i)	Type	Acceptor NBO(j)	Type	E(2)kcal/mol	E(j)-E(i)a.u.	F(i,j)a.u.
C1-C2	σ	C1-N7	σ*	3.57	1.26	0.060
		C2-C9		4.20	1.24	0.064
C1-C3	σ	C2-C9	σ*	4.74	1.20	0.068
		N7-N13		6.09	1.03	0.071
C1-N7	σ	C1-C2	σ*	3.69	1.37	0.064
	π	C2-C9	π*	9.78	0.35	0.056
		C3-O19		12.46	0.36	0.062
C2-C4	σ	C1-N7	σ*	5.09	1.28	0.072
		C2-C9		4.32	1.26	0.066
		C4-C12		4.56	1.25	0.068
C2-C9	σ	C1-C2	σ*	4.44	1.18	0.065
		C2-C4		5.05	1.24	0.071
	π	C1-N7	π*	17.91	0.28	0.064
		C4-C12		21.44	0.28	0.071
		C14-C15		18.82	0.28	0.066
C3-N6	σ	C4-C12	σ*	4.17	1.37	0.068
C3-O19	π	C1-N7	π*	4.17	0.38	0.038
C4-C12	σ	C2-C4	σ*	5.01	1.25	0.071
		C12-C14		4.38	1.27	0.067
		C14-Br21		4.86	0.81	0.056
	π	C2-C9	π*	16.06	0.30	0.062
		C14-C15		21.86	0.29	0.072
C5-C10	σ	C5-C11	σ*	4.11	1.21	0.063
		C11-O22		3.93	1.01	0.056
	π	C8-O20	π*	19.72	0.29	0.068
		C11-C16		22.25	0.28	0.071
		C17-C18		14.31	0.29	0.061
C5-C11	σ	C5-C10	σ*	4.32	1.29	0.067
		C11-C16		4.57	1.28	0.068
C9-C15	σ	C1-C2	σ*	4.61	1.16	0.065
		C2-C9		4.19	1.27	0.065
		C14-C15		5.01	1.25	0.071
		C14-Br21		5.89	0.78	0.061
C9-H29	σ	C2-C4	σ*	4.43	1.05	0.061
		C14-C15		4.46	1.09	0.062
C10-H30	σ	C5-C11	σ*	5.17	1.02	0.065
		C17-C18		4.84	1.05	0.064
C11-C16	σ	C5-C11	σ*	5.34	1.24	0.073
		C16-C18		4.04	1.28	0.064
	π	C5-C10	π*	13.35	0.31	0.058
		C17-C18		17.27	0.31	0.070
C12-C14	σ	C4-N6	σ*	5.21	1.16	0.069
		C4-C12		4.15	1.30	0.066

C12-H31	σ	C2-C4	σ*	4.52	1.06	0.062
		C14-C15		4.63	1.09	0.064
C14-C15	σ	C9-C15	σ*	4.09	1.29	0.065
	π	C2-C9	π*	19.22	0.30	0.069
		C4-C12		17.28	0.29	0.064
C17-C18	π	C5-C10	π*	21.02	0.27	0.071
		C11-C16		16.21	0.26	0.060
		C24-C26		16.87	0.28	0.065
		C25-C27		15.20	0.28	0.062
O22-H34	σ	C5-C11	σ*	4.15	1.28	0.066
		C14-C15		4.88	1.07	0.065
C24-C26	π	C17-C18	π*	15.73	0.29	0.064
		C25-C27		18.67	0.29	0.066
C24-H38	σ	C17-C18	σ*	4.74	1.05	0.063
		C26-C27		4.74	1.05	0.063
C25-C27	σ	C18-C25	σ*	3.54	1.26	0.060
	π	C17-C18	π*	17.66	0.29	0.068
		C24-C26		16.25	0.30	0.063
C25-H39	σ	C17-C18	σ*	4.61	1.06	0.063
C26-H40	σ	C17-C24	σ*	4.49	1.06	0.062
C27-H41	σ	C18-C25	σ*	4.58	1.06	N6.062
N6	LP (1)	C3-O19	π*	48.74	0.29	0.109
		C4-C12		41.50	0.29	0.099
N7	LP (1)	C1-C2	**σ***	13.48	0.83	0.095
		N13-H32		8.15	0.79	0.072
N13	LP (1)	C1-N7	**π***	33.79	0.29	0.092
		C8-O20		46.92	0.30	0.109
O19	LP (2)	C1-C3	**σ***	22.68	0.63	0.108
		C3-N6		28.74	0.66	0.125
O20	LP (2)	C5-C8	σ*	20.30	0.64	0.104
		C8-N13		28.51	0.66	0.124
Br21	LP (2)	C12-C14	σ*	3.04	0.85	0.045
		C14-C15		3.63	0.86	0.050
	LP (3)	C14-C15	π*	9.74	0.31	0.054
O22	LP (1)	C11-C16	σ*	5.08	1.21	0.070
		N13-H32		7.27	1.06	0.078
	LP (2)	C11-C16	π*	25.00	0.38	0.091
