# Supplementary material for: Structure-guided discovery and characterization of novel FLT3 inhibitors for acute myeloid leukemia treatment
Source: PLoS One. 2025 Oct 13;20(10):e0334415. doi: 10.1371/journal.pone.0334415 (PMC12517515; doi:10.1371/journal.pone.0334415)
Supplement: S3 Table — (PDF) [file pone.0334415.s007.pdf]

S3 Table: Second-order perturbation analysis of the interaction between donor and acceptor orbitals of compound Molport-007-550-904 in NBO basis.

Donor NBO(i)	Type	Acceptor NBO(j)	Type	E(2)kcal/mol	E(j)-E(i)a.u.	F(i,j)a.u.
C1-C2	σ	C2-O13	σ*	1.77	1.25	0.42
		N3-C17		4.83	0.98	0.61
		C4-C19		4.72	1.20	0.67
		N7-N11		6.67	1.04	0.75
C1-C4	σ	C1-N7	σ*	4.10	1.26	0.064
		C4-C19		3.96	1.24	0.063
		C6-C20		4.18	1.25	0.065
C1-N7	σ	C1-C2	σ*	1.55	1.31	0.041
		C1-C4		3.82	1.38	0.065
		C8-N11		3.25	1.32	0.059
	π	C1-N7	π*	2.63	0.35	0.028
		C2-O13		12.08	0.36	0.062
		C4-C9		9.10	0.37	0.055
C2 - N3	σ	C1-N7	σ*	1.42	1.38	0.039
		N3 -C6		2.05	1.22	0.045
		C6 -C20		3.87	1.36	0.065
C2-O13	σ	C1-C2	σ*	2.07	1.46	0.050
C2-O13	π	C1-N7	π*	4.17	0.37	0.038
N3-C6	σ	C2-N3	σ*	1.27	1.22	0.036
		C2-O13		3.24	1.41	0.061
		C4-C19		2.38	1.36	0.051
		C6-C20		2.36	1.37	0.051
N3-C17	σ	N3-N6	σ	2.06	1.18	0.044
		C4-C6		1.74	1.27	0.042
C4-C6	σ	C1-C4	σ*	2.24	1.15	0.045
		C1-N7		5.51	1.27	0.075
		N3-C17		4.69	1.03	0.062
		C4-C19		4.39	1.25	0.066
		C6-C20		4.52	1.25	0.068
		C19-H35		2.84	1.11	0.051
C4-C19	σ	C1-C4	σ*	4.40	1.18	0.065
		N3-C6		2.19	1.15	0.045
		C4-C6		5.24	1.24	0.072
		C19-C24		3.32	1.28	0.058
	π*	C1-N7	π*	19.91	0.27	0.066
		C6-C20		21.21	0.29	0.070
		C24-C25		17.10	0.29	0.063
C5-C8	σ	C5-C9 π*	σ*	3.56	1.27	0.060

C5-C9	σ	C5-C10	σ*	4.23	1.19	0.064
		C9-C14		3.56	1.28	0.060
		C10-O18		3.26	1.09	0.053
		C14-C21		3.01	1.25	0.055
	π	C8-O10	π*	30.83	0.24	0.078
		C10-C12		19.92	0.30	0.069
C5-C10	σ	C5-C9 π*	σ*	4.12	1.25	0.064
		C8-N11		3.33	1.06	0.054
		C10-C12		3.65	1.26	0.061
C6-C20	σ	C4-C6	σ*	5.06	1.24	0.071
		C20-C25		3.36	1.28	0.059
	π	C4-C19	π*	16.26	0.29	0.062
		C24-C25		22.59	0.29	0.073
C9-C14	σ	C5-C8	σ*	3.75	1.11	0.058
		C14-C15		4.03	1.23	0.063
C9-H29	σ	C5-C10	σ*	4.95	1.00	0.063
		C14-C15		5.05	1.05	0.065
C10-C12	σ	C5-C10	σ*	3.92	1.19	0.061
		C12-C15		3.70	1.28	0.061
	π	C5-C9	π*	13.58	0.28	0.056
N11-H30	σ	C5-C8	σ*	3.61	1.13	0.058
C12-C15	σ	C14-C15	σ*	3.77	1.22	0.061
C12-H31	σ	C5-C10	σ*	5.01	0.99	0.063
		C14-C15		4.79	1.05	0.063
C14-C15	σ	C9-C14	σ*	3.69	1.23	0.060
		C14-C21		3.60	1.21	0.059
C14-C21	σ	C9-C14	σ*	3.86	1.24	0.062
		C14-C15		3.74	1.21	0.060
C15-C22	σ	C12-C15	σ*	3.60	1.24	0.060
C17-H33	σ	N3-C6	σ*	5.05	0.94	0.062
O18-H34	σ	C10-C12	σ*	4.89	1.32	0.072
C19-H24	σ	C1-C4	σ*	5.19	1.17	0.070
C19-H35	σ	C4-C6	σ*	4.54	1.06	0.062
C20-C25	σ	N3-N6	σ*	5.96	1.13	0.074
C20-H36	σ	C4-C6	σ*	4.84	1.05	0.064
C21-C28	σ	C14-C21	σ*	3.34	1.25	0.058
	π	C22-C27	π*	18.65	0.30	0.066
C21-H37	σ	C14-C15	σ*	4.72	1.05	0.063
C22-C27	σ	C15-C22	σ*	3.46	1.26	0.059
	π	C21-C28	π*	15.61	0.30	0.061
C22-H38	σ	C14-C15	σ*	4.56	1.05	0.062
C24-C25	π	C4-C19	π*	22.61	0.28	0.072
		C6-C20		16.79	0.28	0.062
C24-H41	σ	C4-C19	σ*	4.24	1.09	0.061
C25-H42	σ	C6-C20	σ*	4.06	1.10	0.060

N3	LP (1)	C2-O13	π*	54.12	0.28	0.112
		C6-C20		42.81	0.28	0.098
N7	LP (1)	C1-C4	σ*	12.80	0.85	0.094
N11	LP (1)	C1-N7	π*	37.70	0.29	0.096
		C8-O16		51.82	0.28	0.108
O13	LP (2)	C1-C2	σ*	21.99	0.64	0.108
		C2-N3		27.07	0.68	0.123
C14	LP (1)	C5-C9	π*	84.40	0.13	0.112
		C21-C28		56.67	0.14	0.101
O16	LP (2)	C5-C8	σ*	11.28	0.74	0.083
		C8-N11		21.92	0.70	0.112
		O18-H34		19.29	0.73	0.108
O18	LP (1)	C5-C10	σ*	8.04	1.06	0.082
	LP (2)	C10-C12	π*	35.86	0.34	0.102
