# Supplementary material for: Structure-guided discovery and characterization of novel FLT3 inhibitors for acute myeloid leukemia treatment
Source: PLoS One. 2025 Oct 13;20(10):e0334415. doi: 10.1371/journal.pone.0334415 (PMC12517515; doi:10.1371/journal.pone.0334415)
Supplement: S4 Table — (PDF) [file pone.0334415.s008.pdf]

S4 Table: Mulliken and Natural charges of all the atoms in MolPort-007-550-904 and MolPort-002-705-878 molecules.

**Atoms of MolPort-007-550-904**	**Mulliken charges for MolPort-007-550-904**	**Natural charges for MolPort-007-550-904**	**Atoms of MolPort-002-705-878**	**Mulliken charges for MolPort-002-705-878**	**Natural charges for MolPort-002-705-878**
C1	0.068716	0.11626	C1	-0.012779	0.09953
C2	0.428234	0.68362	C2	-0.08277	-0.13782
N3	-0.517527	-0.45981	C3	0.39672	0.67633
C4	-0.101593	-0.14344	C4	0.24141	0.21741
C5	-0.293929	-0.23351	C5	-0.230521	-0.19226
C6	0.271755	0.22075	N6	-0.495991	-0.61729
N7	-0.192721	-0.20910	N7	-0.120937	-0.15896
C8	0.530469	0.69732	C8	0.497059	0.68211
C9	0.038490	-0.10620	C9	-0.017922	-0.16530
C10	0.203963	0.38884	C10	0.011402	-0.09650
N11	-0.292988	-0.37843	C11	0.202887	0.35137
C12	-0.065163	-0.25799	C12	0.035656	-0.27304
O13	-0.335211	-0.58370	N13	-0.342662	-0.41759
C14	-0.106358	-0.08648	C14	-0.191111	-0.04624
C15	-0.010996	-0.01612	C15	-0.062872	-0.07397
O16	-0.422602	-0.64951	C16	-0.057371	-0.27046
C17	-0.076863	-0.17646	C17	-0.064478	-0.07582
O18	-0.357808	-0.67174	C18	-0.050274	-0.02374
C19	-0.048919	-0.16487	O19	-0.295504	-0.55213
C20	-0.063085	-0.25510	O20	-0.308311	-0.56534
C21	-0.049889	-0.15247	Br21	-0.021068	0.05726
C22	-0.066952	-0.19355	O22	-0.429384	-0.71175
C23	-0.222670	-0.37462	C23	-0.256448	-0.58249
C24	-0.094463	-0.22195	C24	-0.041814	-0.15570
C25	-0.085509	-0.15608	C25	-0.065941	-0.19071
C26	-0.301953	-0.56848	C26	-0.092787	-0.20424
C27	-0.079999	-0.17384	C27	-0.080135	-0.17570
C28	-0.099169	-0.21779	H28	0.234754	0.41022
H29	0.149235	0.24413	H29	0.088805	0.20499
H30	0.225580	0.37810	H30	0.116296	0.23437
H31	0.090826	0.21600	H31	0.111699	0.22621
H32	0.120540	0.18825	H32	0.281323	0.39805
H33	0.142658	0.21836	H33	0.079944	0.20054
H34	0.264133	0.49063	H34	0.272516	0.48153

**Atoms of MolPort-007-550-904**	**Mulliken charges for MolPort-007-550-904**	**Natural charges for MolPort-007-550-904**	**Atoms of MolPort-002-705-878**	**Mulliken charges for MolPort-002-705-878**	**Natural charges for MolPort-002-705-878**
H35	0.090561	0.19354	H35	0.093385	0.19665
H36	0.108095	0.21365	H36	0.140534	0.21732
H37	0.101297	0.20979	H37	0.135991	0.21422
H38	0.084295	0.20208	H38	0.095726	0.21422
H39	0.128045	0.19931	H39	0.084119	0.20148
H40	0.112272	0.18856	H40	0.100388	0.20586
H41	0.100247	0.20849	H41	0.100463	0.20414
H42	0.104091	0.20605	-	-	-
H43	0.117383	0.20268	-	-	-
H44	0.113498	0.19365	-	-	-
H45	0.103192	0.18808	-	-	-
H46	0.095293	0.20058	-	-	-
H47	0.093499	0.20255	-	-	-
